# Identification of Semicarbazones, Thiosemicarbazones and Triazine Nitriles as Inhibitors of *Leishmania mexicana* Cysteine Protease CPB

**DOI:** 10.1371/journal.pone.0077460

**Published:** 2013-10-16

**Authors:** Jörg Schröder, Sandra Noack, Richard J. Marhöfer, Jeremy C. Mottram, Graham H. Coombs, Paul M. Selzer

**Affiliations:** 1 Molecular Discovery Sciences, MSD Animal Health Innovation GmbH, Schwabenheim, Germany; 2 Wellcome Trust Centre for Molecular Parasitology, Institute of Infection, Immunity and Inflammation, College of Medical, Veterinary and Life Sciences, University of Glasgow, Glasgow, United Kingdom; 3 Strathclyde Institute of Pharmacy and Biomedical Sciences, University of Strathclyde, Glasgow, United Kingdom; 4 Interfakultäres Institut für Biochemie, University of Tübingen, Tübingen, Germany; Technion-Israel Institute of Technology Haifa 32000 Israel., Israel

## Abstract

Cysteine proteases of the papain superfamily are present in nearly all eukaryotes. They play pivotal roles in the biology of parasites and inhibition of cysteine proteases is emerging as an important strategy to combat parasitic diseases such as sleeping sickness, Chagas’ disease and leishmaniasis. Homology modeling of the mature *Leishmania mexicana* cysteine protease CPB2.8 suggested that it differs significantly from bovine cathepsin B and thus could be a good drug target. High throughput screening of a compound library against this enzyme and bovine cathepsin B in a counter assay identified four novel inhibitors, containing the warhead-types semicarbazone, thiosemicarbazone and triazine nitrile, that can be used as leads for antiparasite drug design. Covalent docking experiments confirmed the SARs of these lead compounds in an effort to understand the structural elements required for specific inhibition of CPB2.8. This study has provided starting points for the design of selective and highly potent inhibitors of *L. mexicana* cysteine protease CPB that may also have useful efficacy against other important cysteine proteases.

## Introduction

Current drug therapy for the treatment of neglected diseases associated with parasitic protozoa mainly relies on drugs developed decades ago. Severe toxic effects combined with the emergence of drug resistant parasite strains create an urgent and continuous need for new, safe and effective drugs against leishmaniasis [1,2]. Cysteine proteases constitute a pivotal class of enzymes that play numerous roles in the biology of these trypanosomatid parasites [3,4]. Identification and further characterization of cysteine protease-mediated processes in parasitic protozoa is progressing [5-7] and supporting the idea that a possible strategy for combating parasitic infections is to inhibit cysteine proteases that are crucial to parasite metabolism and reproduction. Papain-like cysteine proteases have been identified in *T. cruzi* (cruzain) [8], *T. brucei* (trypanopain, TbCatB) [9] and different *Leishmania* spp. (CPA, CPB, CPC) [10,11] and inhibition of these peptidases has led to promising results both *in vitro* [12], in tissue culture models [13-15] and *in vivo* [15-17]. This study has focused on finding inhibitors of CPB, a cathepsin L-like cysteine protease thought to be crucial in the infectivity of *Leishmania mexicana* and encoded as a tandem array of 19 similar genes [18]. CPB expression is regulated so that CPB1 and CPB2, the first two genes of the tandem array, are expressed in the infectious metacyclic stage and the remaining genes in the intracellular amastigote stage that causes the disease [19]. Due to their high sequence identity [20], the multiple isoforms present in amastigotes are expected to have similar inhibitor susceptibilities. A recombinant form of the amastigote-specific isoform CPB2.8, expressed without the C-terminal extension and so designated CPB2.8∆CTE [21], was used in this study.

Inhibitors of cysteine proteases typically rely on the presence of a warhead, an electrophilic functionality that is attacked by the catalytic cysteine thiolate in the active site of the target enzyme [22-24]. Inhibitors containing a reversible reactive warhead-type might be expected to possess better safety profiles with regards to their potential application as drugs for treating parasitic infections, examples of such reactive inhibitors of *L. mexicana* CPB are compounds of the class of α-ketoheterocycles [25]. In order to identify new warhead-types that are reversibly reactive and have some specificity for cysteine proteases of trypanosomatid parasites, high throughput screening of a compound library against *L. mexicana* CPB2.8 and bovine cathepsin B as a counter assay was performed. Homology modeling and covalent docking studies to rationalize the experimental findings were also carried out. Thus, it was established that semicarbazones, thiosemicarbazones and triazine nitriles are competitive inhibitors of *L. mexicana* CPB2.8∆CTE. 

## Results

### Homology modeling of *L. mexicana* CPB2.8∆CTE

In order to get the protein’s 3D structure for covalent docking studies and subsite residue determination, a structural model of mature *L. mexicana* CPB2.8∆CTE using comparative modeling was generated [26]. Due to its excellent structure resolution of 1.75 Å and low B-factors [27], cruzain (PDB ID 1EWP) co-crystallized with the irreversible fluoromethyl ketone inhibitor Mor-Leu-Hpq was used as a template [28]. The mature protein full-length sequence identity of 60% and sequence similarity of 74% between CPB2.8∆CTE and cruzain was reasonable for the generation of a qualified homology model. The resulting homology model of CPB2.8∆CTE showed a Cα RMSD value of 0.699 Å compared to its template structure. The homology model was then structurally compared to bovine cathepsin B (BtCatB, PDB ID 1QDQ) by superimposing the two protein structures. The locations of the amino acids that differ between *L. mexicana* CPB and BtCatB are given in Table 1. Comparison of the active sites indicates high residue similarity for the S_1_’ and the catalytic triade but also a significant residue difference between CPB2.8∆CTE and BtCatB in the S_2_ subsite (Y210E; numbering is according to the mature CPB2.8 enzyme, Table 1). This difference in the S_2_ subsite of the parasite and the host enzyme provides optimism for lead compound optimization approaches for the development of selective inhibitors that target the parasite protein. 

**Table 1:** Key active site residues of *L. mexicana* CPB2.8ΔCTE, BtCatB, and for comparison human cathepsin B (HsCatB).

**Protease^a^**	**S2 Subsite**	**S1 Subsite**	**Catalytic triade**	**S1’ Subsite**
CPB2.8ΔCTE	L70, **Y210**,V212	L68, M69, A140, G165	C26, H164, N184	Q20, W186
BtCatB	S77, **E245**, V247	E75, P76, A173, A200	C29, H199, N219	Q23, W221
HsCatB	A77, **E245**, V247	Y75, P76, A173, A200	C29, H199, N219	Q23, W221

[a] Numbers refer to the mature sequences.

Y210 from CPB2.8ΔCTE and E245 from CatB are highlighted in bold.

### HTS of recombinant CPB2.8∆CTE for inhibitors

**Figure 1:**
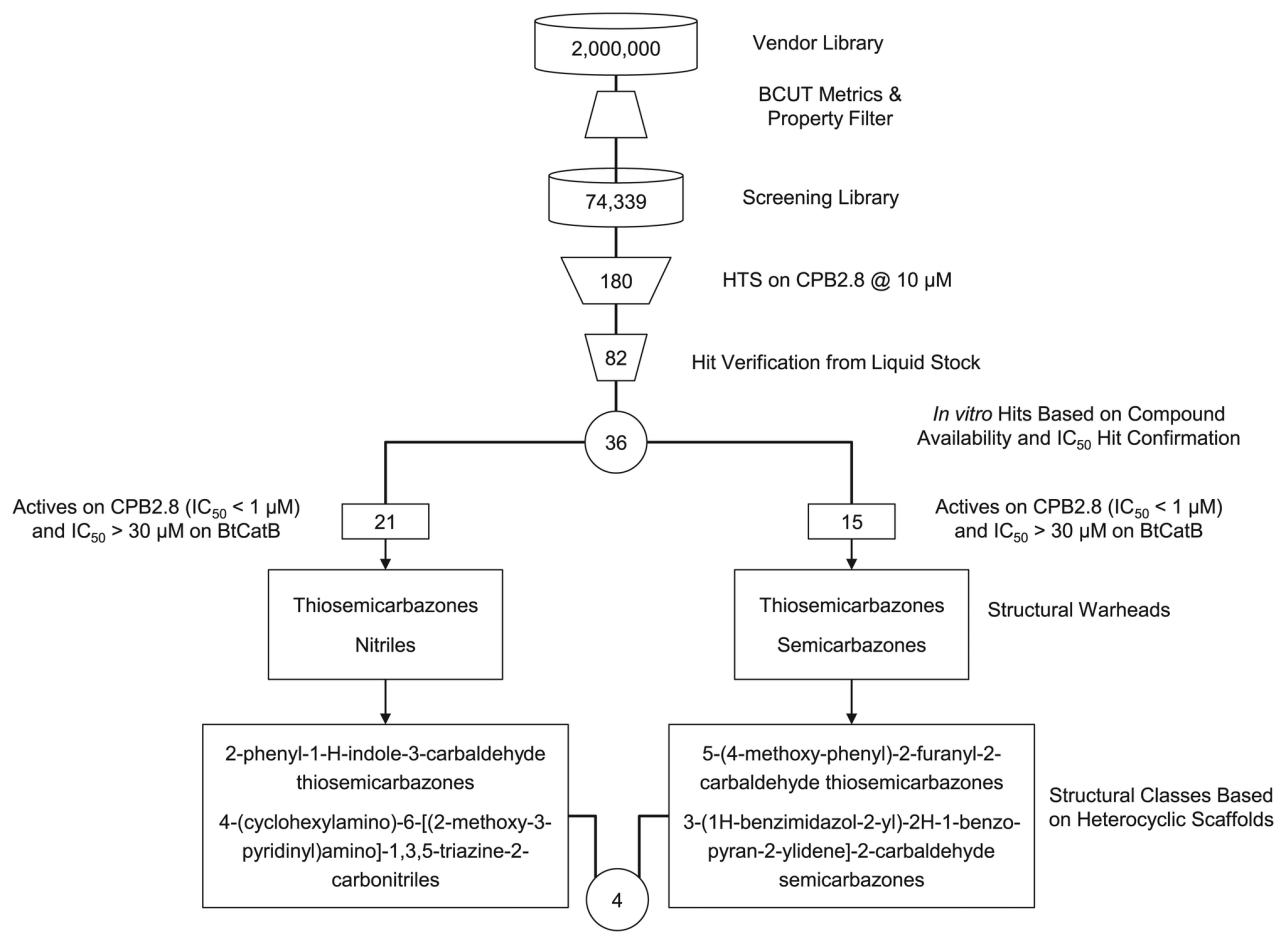
Discovering the lead compounds against *L*. ***mexicana* CPB2.8∆CTE**. Set up of the screening library and filtering steps during hit enrichment. Four confirmed leads were finally identified by the hit enrichment workflow: one of the warhead-type semicarbazone, two of the warhead-type thiosemicarbazone and one of the warhead-type triazine nitrile.

To identify inhibitors of CPB2.8∆CTE that could be lead candidates for further optimization in an antiparasitic drug discovery program, a screening library in a competition assay involving Z-F-R-AMC as a substrate was tested. The library was set up as depicted in Figure 1. Starting from a database of 2 million commercially available compounds, we used the molecular descriptor BCUT metrics developed from the work of Burden and Pearlman [29-31] to select a structurally diverse subset. This subset was further filtered by application of a property filter (see Experimental Section for details), which decreased the number of compounds in the library to 74,339 entities. This library was used to screen CPB2.8∆CTE at single compound concentration of 10 µM. During hit verification (IC_50_ determination from liquid compound stock) BtCatB was used in a counter assay to provide information on the selectivity profile of the inhibitors. We used BtCatB in the counter assay for practical reasons because in parallel to this study we ran a screening on *Eimeria tenella* Cathepsin B-like enzyme also using BtCatB in the counter screen [23]. 

This assay resulted in 82 verified hits exhibiting an IC_50_ ≤ 30 µM on CPB2.8∆CTE. These hits were then subjected to an *in vitro* hit confirmation procedure. Thus, IC_50_ determination was replicated by using freshly dissolved compound from either solid stock taken from the supplier or in-house resynthesized solid stock, in order to eliminate potential false positives caused by, e.g., degradation products. To confirm the actual molecular structure in the test tube with the molecular structure stored in the database, the relevant hit compounds were analyzed by NMR spectroscopy and LCMS. Subsequently, the kinetic aqueous solubility of the hits was determined using nephelometry. In summary, 36 compounds could be ordered in sufficient amounts from the suppliers. All of these 36 confirmed hits passed the solubility criteria (solubility up to 30 µM) and the quality control criteria.

**Figure 2:**
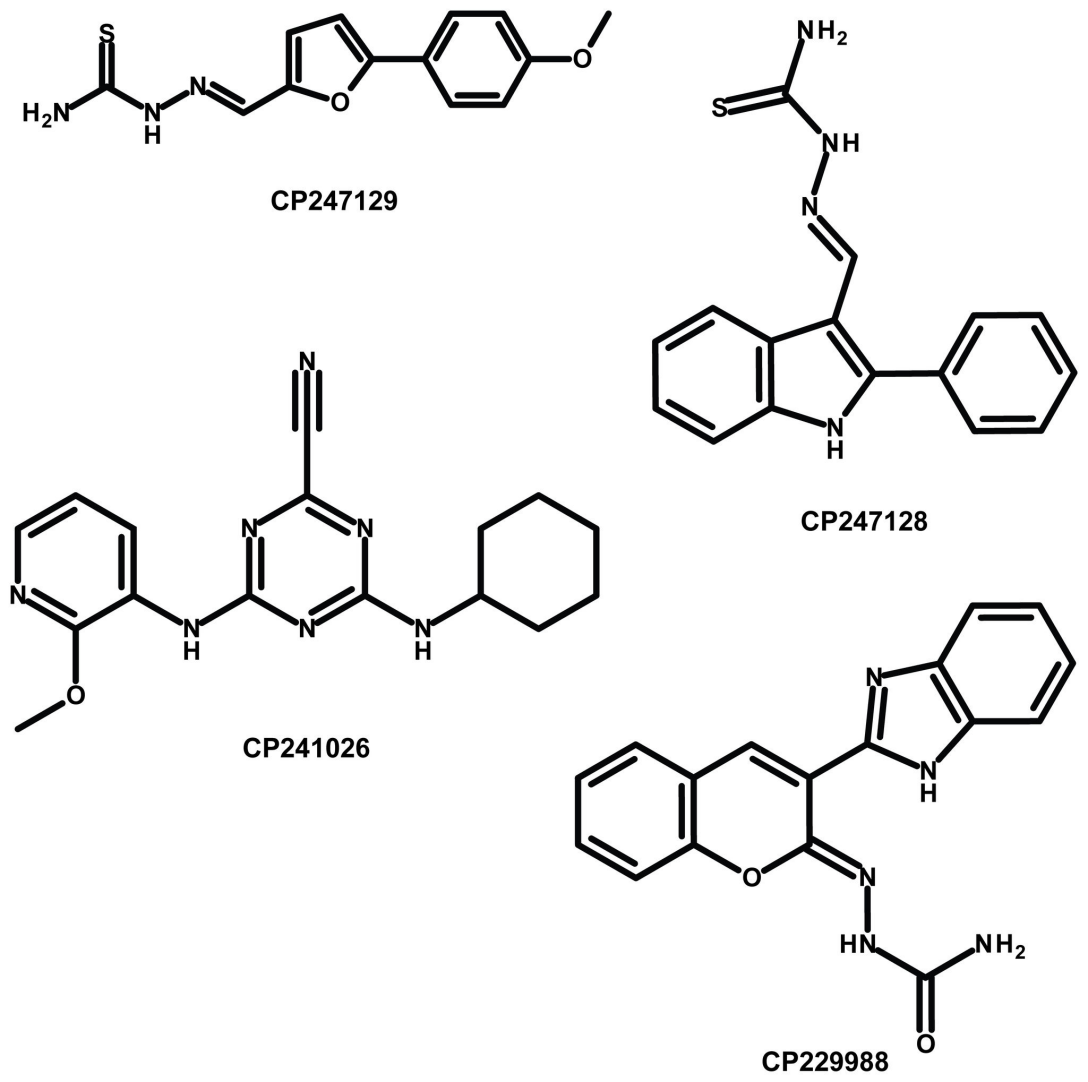
Confirmed lead structures inhibiting CPB2.8∆CTE of *L*. ***mexicana***. Two thiosemicarbazones (CP247129 and CP247128), one nitrile (CP241026) and one semicarbazone (CP229988).

Biochemical results of the tested compounds revealed a broad activity range (IC_50_) against CPB2.8∆CTE, from a high micromolar range down to double-digit nanomolar potency. A total of 15 compounds exhibited IC_50_ values below 1 µM on CPB2.8∆CTE while they showed no activity on BtCatB (IC_50_ > 30 µM). The remaining 21 compounds also showed IC_50_ values below 1 µM on CPB2.8∆CTE and were active on BtCatB as well (IC_50_ ≤ 30 µM). While thiosemicarbazones were present in both groups, semicarbazones were exclusively found in the selective group (IC_50_ < 1 µM on CPB2.8∆CTE and IC_50_ > 30 µM on BtCatB) and nitriles were exclusively found in the less selective group (IC_50_ < 1 µM on CPB2.8∆CTE and IC_50_ ≤ 30 µM on BtCatB). *K*_i_ values were calculated for the four most active compounds, which were also qualified as confirmed leads. Structures (Figure 2), warhead-type, IC_50_ and *K*_i_ values toward CPB2.8∆CTE are presented in Table 2 together with the data toward BtCatB. 

**Table 2:** Confirmed lead structures inhibiting CPB2.8∆CTE of *L. mexicana*.

Compound	Warhead-Type	IC50 [µM]		*K*i [µM]^a^
		CPB2.8ΔCTE	BtCatB	CPB2.8ΔCTE
**CP247129**	Thiosemicarbazon	0.07 ± 0.04	>30	0.04 ± 0.02
**CP247128**	Thiosemicarbazon	0.06 ± 0.01	0.02 ± 0.004	0.04 ± 0.02
**CP241026**	Nitrile	1.1 ± 0.15	13.8 ± 4.5	0.57 ± 0.08
**CP229988**	Semicarbazone	0.01 ± 0.003	>30	0.005 ± 0.002

[a] assuming compounds are competitive inhibitors.

Two thiosemicarbazones, one nitrile and one semicarbazone. BtCatB was used in the counter assay to test the selectivity profile. Results are expressed as mean IC_50_ ± SD from three independent experiments and as calculated *K*_i_ ± SD using the Cheng-Prusoff equation [29].

The leads belong to three different warhead-types, namely thiosemicarbazones (**CP247129**, **CP247128**), nitriles (**CP241026**) and semicarbazones (**CP229988**). The thiosemicarbazone **CP247129** displayed an IC_50_ on CPB2.8∆CTE in the nanomolar range while the compound was inactive on BtCatB (IC_50_ > 30 µM). Conversely, the thiosemicarbazone (**CP247128**) exhibited an IC_50_ on both CPB2.8∆CTE and on BtCatB in the nanomolar range (Table 2). The nitrile **CP241026** was approximately ten times less active on CPB2.8∆CTE (*K*_i_ = 570 nM) compared to both thiosemicarbazones but exhibited weak activity on BtCatB (IC_50_ = 13.8 µM). The most potent and specific CPB2.8∆CTE inhibitor identified in the assay was the semicarbazone **CP229988** with a *K*_i_ of 5 nM and an IC_50_ > 30 µM toward BtCatB. 

### Covalent Docking Analysis

**Figure 3:**
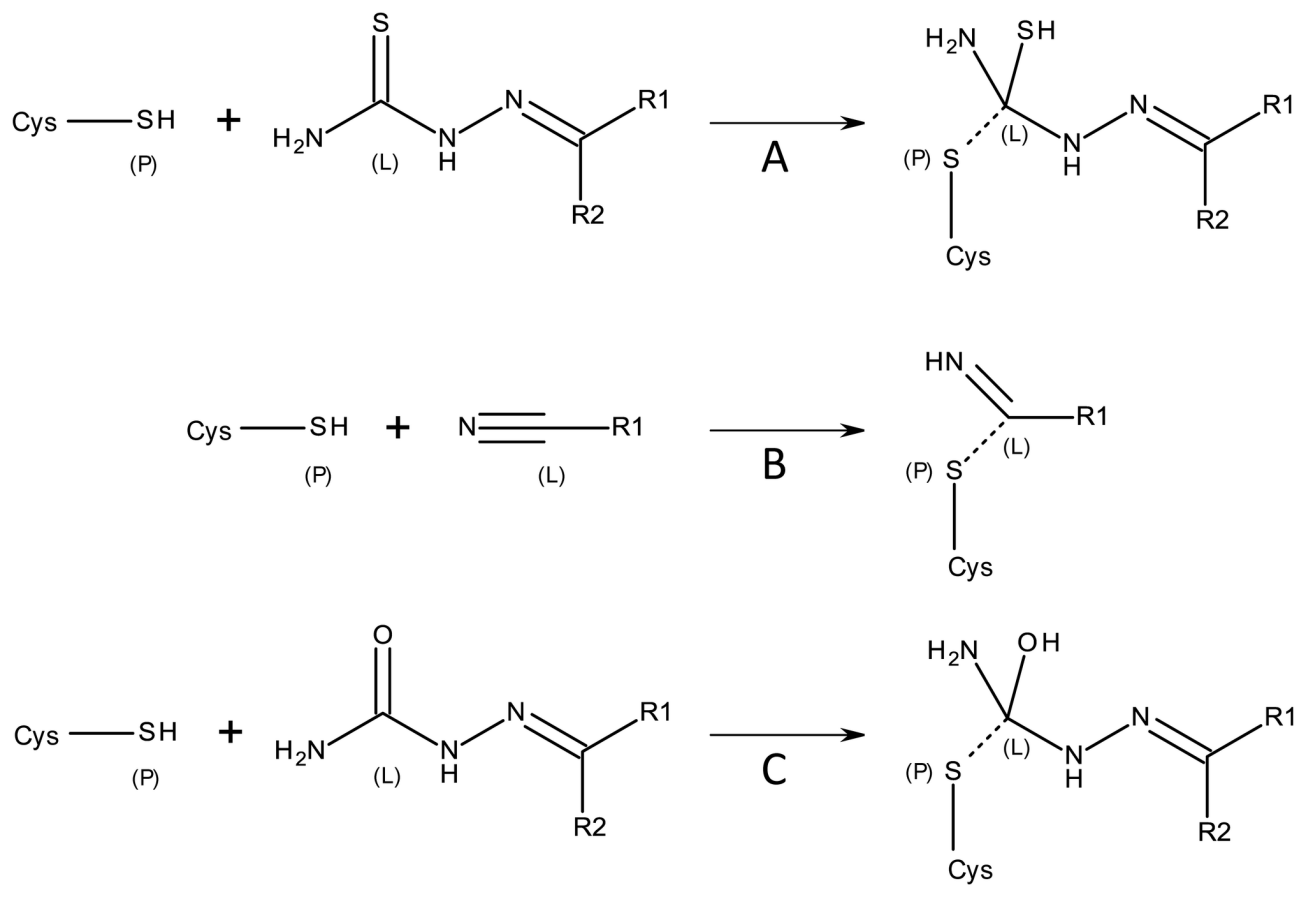
Schematic representation of the covalent binding of warheads for the covalent docking process. Atoms that form the covalent bond are labeled P in the protein and L in the ligand. Covalent bonds between P and L are indicated with dashed lines. (a-d) Reaction between thiosemicarbazone[30], nitrile[31] and semicarbazone warhead of the ligand and the catalytic cysteine residue of the protein.

**Figure 4:**
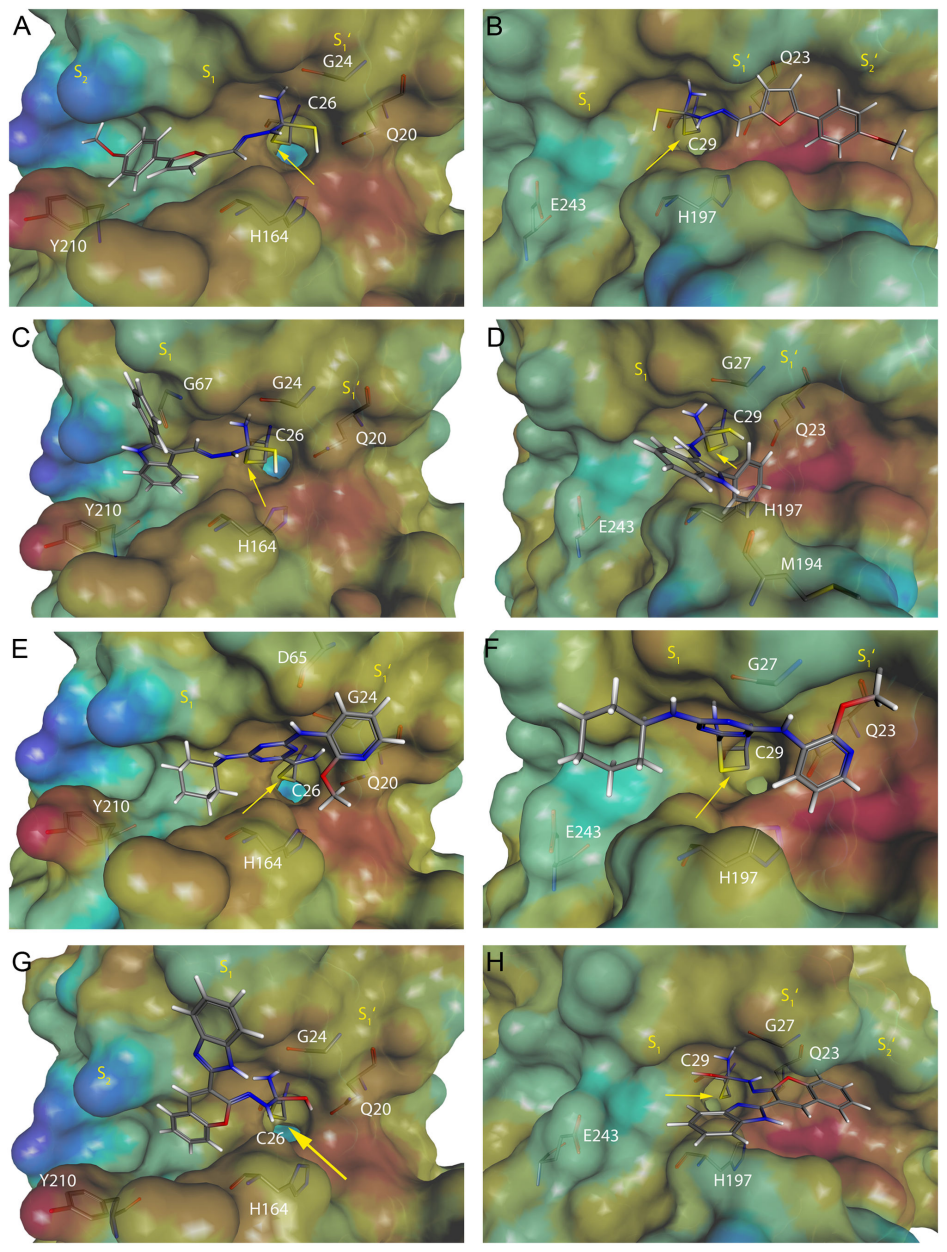
Covalent docking solutions of thiosemicarbazone-, nitrile- and semicarbazone-based inhibitors in the active site of the *L*. ***mexicana* CPB2.8∆CTE homology model (A, C, E, G) and the X-ray structure of BtCatB (B, D, F, H)**. The transparent solvent accessible surface of the active sites is colored by lipophilic potential. The color ramp ranges from brown (highest lipophilic area of the surface) to blue (highest hydrophilic area of the surface). The relevant cysteine residue has been excluded from surface generation for visibility reasons of the covalent bond between the enzyme and the inhibitor molecule. Relevant amino acids of the active sites and the corresponding inhibitors are depicted in capped-stick representation and colored by atom type. The yellow arrow marks the covalent bond between the catalytic cysteine and the inhibitor. **A**: **CP247129** covalently docked into the active site of CPB2.8∆CTE, **B: CP247129** covalently docked into the active site of BtCatB, **C: CP247128** covalently docked into the active site of CPB2.8∆CTE, **D: CP247128** covalently docked into the active site of BtCatB, **E**: CP241026 covalently docked into the active site of CPB2.8∆CTE, **F: CP241026** covalently docked into the active site of BtCatB, **G: CP229988** covalently docked into the active site of CPB2.8∆CTE, **H: CP229988** covalently docked into the active site of BtCatB.

Covalent docking [24] as implemented in GOLD 5.0.1 was employed to predict the protein/ligand binding interactions of the four lead structures using the previously generated homology model of mature CPB2.8∆CTE and the publicly available X-ray crystal structure of BtCatB (PDB ID 1QDQ) [33]. The basic chemical reactions of the warheads proposed for covalent docking are shown in Figure 3. The accordingly modified compounds were docked independently into the binding site of both cysteine proteases (see Experimental Section for details). In order to visualize surface regions, the program MOLCAD [34] was applied. Compound **CP247129** was selected for covalent docking studies due to its differences in inhibitory activity on CPB2.8∆CTE (*K*_i_ = 40 nM) compared to BtCatB (IC_50_ > 30 µM). As expected, a significant difference between the orientation of the top ranked poses of **CP247129** covalently bound to the active site of CPB2.8∆CTE (Figure 4A) and BtCatB (Figure 4B) was observed. In Figure 4A the 4-methoxy phenyl portion of **CP247129** is oriented toward the deep S2 subsite and the furanyl moiety occupies the shallow S1 subsite. The thiosemicarbazone scaffold was assumed to interact via a 1,2-polar addition of the catalytic C26 to the C=S group of **CP247129** (Figure 3A) [23,35]. Thus, the resulting tetrahedral transition state was used as the initial conformation of the thiosemicarbazone scaffold for covalent docking. The thiole group represents a prochiral center and as it is *a priori* not known which isomer resembles the most active transition state, both possible stereo isomers were generated for the covalent docking procedure. In Figure 4A the top ranked S-isomer of **CP247129** is shown, and the covalent bond between the C26 sulfur and the transformed thiocarbonyl carbon is marked by a yellow arrow. The NH2 group of the thiosemicarbazone scaffold fits close to the carbonyl of G24. The distance between the nitrogen and the oxygen of the G24 carbonyl was calculated to be 2.18 Å, supporting a hydrogen bond between the two atoms. The reaction of C26 to the C=S group would be further assisted by the transfer of the H164 proton to the thiosemicarbazone sulfur. Figures 4A to 4H also show the MOLCAD lipophilic potential (LP) surfaces of the corresponding binding sites. The color for LP ranges from brown (highest lipophilic area of the surface) to blue (highest hydrophilic area of the surface). As depicted in Figure 4A, the 4-methoxy-phenyl-2-furanyl portion of **CP247129** is oriented to a brown region, suggesting that hydrophobic substituents may be favored in the S_1_ and S_2_ subsite of CPB2.8∆CTE. In contrast, the 4-methoxy-phenyl-2-furanyl portion of **CP247129** covalently bound to the C29 thiolate of BtCatB is oriented to the primed subsites as depicted in Figure 4B. The unprimed subsites of BtCatB show a green LP region, suggesting that hydrophilic groups would be favorable due to the Y210E/L70S exchange in the S_2_ subsite. Figure 4B clearly shows that key hydrogen bonding and hydrophobic contacts that are established in the complex of the active S-enatiomer with CPB2.8∆CTE are completely disrupted. These observations could explain the lack of potency of CP247129 toward BtCatB.

A similar binding mode to CPB2.8∆CTE could be observed for the top ranked S-isomer of **CP247128** when compared to **CP247129** (Figure 4C). The hydrophobic 2-phenyl-1H-indole portion occupies the S_1_ and S_2_ subsite while the nitrogen of the indole is oriented near the carbonyl oxygen of G67. The calculated distance of 2.88 Å accounts for a stabilizing hydrogen bond. Furthermore, probably stabilizing hydrogen bonds of the thiolate intermediate can be established as described for **CP247129**. The MOLCAD lipophilic potential predicts a brown region for the S_1_ and S_2_ subsites, which satisfactorily match the hydrophobic 2-phenyl-1H-indole portion of **CP247128**. An unexpected binding mode was observed for the top ranked S-isomer of **CP247128** covalently docked into the active site of BtCatB (Figure 4D). Due to the green LP region of the first two unprimed subsites of BtCatB, the phenyl portion of **CP247128** was placed into the S_1_’ subsite while the nitrogen of the indole moiety fits close to the oxygen of the M194 carbonyl with a calculated distance of 3.09 Å. This covalent docking solution of **CP247128** allows key hydrogen bonds of the tetrahedral transition state, and thus could explain the nanomolar potency of **CP247128** toward both CPB2.8∆CTE and BtCatB. 

Figure 4E shows the top ranked pose of the triazine nitrile **CP241026** covalently bound to the active site of CPB2.8∆CTE. A thioimidate moiety formed by covalent interaction between the nitrile warhead and the C26 thiolate in the CPB2.8∆CTE catalytic site was assumed and generated for the covalent docking procedure (Figure 3B) [36]. The cyclohexyl group is placed into the hydrophobic S_2_ subsite while the 2-methoxy-pyridyl moiety occupies the S_1_’ subsite. The 3-pyridyl secondary amino group of **CP241026** is oriented close to the oxygen of the D65 carbonyl group, suggesting a hydrogen bond between N and O with a calculated distance of 2.41 Å. A different binding mode was observed for the top ranked covalent docking solution of **CP241026** bound to the active site of BtCatB (Figure 4F). The green LP surface of the S_2_ subsite forces the hydrophobic cyclohexyl group to twist, which resulted in unfavorable BtCatB interactions and probably weak inhibitory activity of **CP241026** toward the enzyme. 

**Figure 5:**
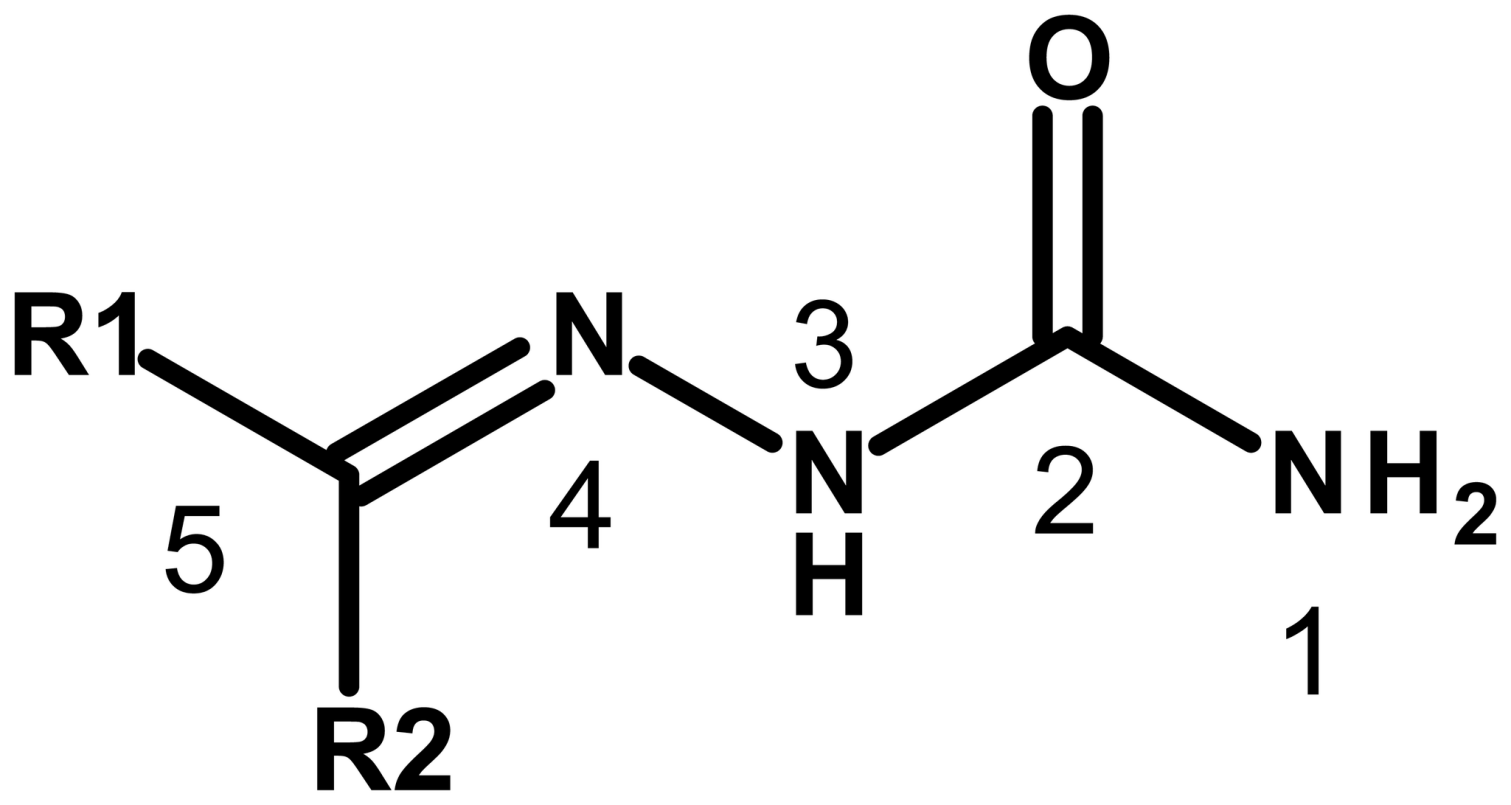
Semicarbazone scaffold and numbering of atoms.

Although, it seems to be accepted that peptidyl semicarbazones inhibit cysteine proteases through the formation of a reversible tetrahedral adduct by attack of the thiolate on the C-5 carbon[37] (Figure 5) covalently docking the semicarbazone **CP229988** into the active site of the CPB2.8∆CTE homology model and the BtCatB X-ray structure using the tetrahedral adduct of the thiolate and the C-5 carbon as the initial docking conformation results in poor docking scores only. This was also true for the parent ketone benzopyran-2-on of **CP229988**. Thus, the only logical site of covalent interaction with CPB2.8∆CTE in **CP229988** is the C-2 double bond (Figure 5) [38]. Indeed, using the C-2 carbon for the docking led to a docking solution with a reasonable docking score as shown in Figure 4G, which shows the top ranked pose of the semicarbazone **CP229988** covalently bound to the active site of CPB2.8∆CTE. A thiohemiketal between the carbonyl group of the semicarbazone scaffold and the C26 thiolate in the CPB2.8∆CTE catalytic site was assumed (Figure 3C) [38]. Because the carbonyl group represents a prochiral center, both possible stereoisomers were generated for the covalent docking procedure. In Figure 4G the top ranked S-isomer of **CP229988** is shown, and the covalent bond between the C26 sulfur and the C-2 carbon is marked by a yellow arrow. The amino group of the semicarbazone scaffold fits close to the carbonyl of G24 and the calculated distance of 2.18 Å suggests a stabilizing hydrogen bond. The reaction of C26 to the carbonyl group would be further assisted by the transfer of the H164 proton to the semicarbazone oxygen. The benzopyran-2-ylidene portion of **CP229988** is oriented to the hydrophobic S_2_ subsite, while the benzimidazol-2yl moiety occupies the S_1_ subsite of CPB2.8∆CTE. In contrast, the (1H-benzimidazol-2-yl)-2H-1-benzopyran-2-ylidene portion of **CP229988** covalently bound to the C29 sulfur of BtCatB is oriented to the primed subsites as depicted in Figure 4H. No top ranked covalent docking pose could be obtained for **CP229988** and BtCatB. In addition, Figure 4H clearly shows that key hydrogen bonding and hydrophobic contacts that are established in the tetrahedral complex of the active S-enantiomer with CPB2.8∆CTE are completely disrupted. 

## Discussion

In this study, 74,339 structurally diverse compounds coming from a general screening library have been tested as inhibitors of a recombinant form of the cathepsin L-like cysteine protease CPB present in the parasite *L. mexicana* (CPB2.8∆CTE). In a separate assay, the compounds were evaluated for their ability to inhibit cathepsin B from bovine spleen. BtCatB was chosen over the higly similar HsCatB because of easy accessibility. Two unexpected results emerge from this study. First, molecules with novel cysteine protease warheads were not identified in this study. This is startling, given the effort devoted to identify inhibitors bearing covalent reversible warheads in screening libraries [24]. Because the structurally diverse screening set was filtered from a general purpose screening library of 2,000,000 compounds, we assumed to find more suitable warheads. Even taking into account the lack of target bias in the chemotypes represented in it, the result suggests that potent covalent reversible inhibition of CPB2.8∆CTE is limited to only a few warheads. Second, only compounds from the thiosemicarbazone and semicarbazone warhead-type were identified as specific, reversible inhibitors of CPB2.8∆CTE but with no activity against BtCatB (IC50 > 30 µM); the selectivity ratio (CPB2.8∆CTE/BtCatB) was <0.00033 for the most active inhibitor CP229988. These two findings will be considered in turn.

The lack of novel covalent reversible acting warheads identified in the CPB2.8∆CTE HTS highlights the limitations of screening a relatively small and unbiased library within a large chemical possibility space. The screening library was designed for general use, with no single family of targets in mind, and it is not dominated by any one chemotype. Finding new chemotypes from libraries for which there is no ligand bias for the protein is an ongoing challenge in the field [39,40]. This explains the good track record of HTS against chemically well-explored targets such as G-protein coupled receptors and kinases and its often limited results against new genomic targets [41]. Expanding the library by even an order of magnitude, however, and adding chemotypes from different sources, will only partially address this chemical space problem [42,43]. Another solution would be to simply screen at higher concentrations of compound, but this presents logistical difficulties, including exhaustion of source material, insolubility of compound in the assay, and an increase in the number of artifactual hits. An alternative is to prioritize a small subset of more likely chemotypes for careful testing, often at higher concentrations. One approach to do this is by looking for complementary fits of particular library molecules to the structure of the target, as is afforded by molecular or covalent docking [24]. However, our findings using the unbiased library provide good evidence that the three warhead types found to inhibit are perhaps those compound groups most worth pursing in the search for drugs against these cysteine proteases. 

We used in this study covalent docking to study the binding mode of the newly identified inhibitors with their protein targets CPB2.8∆CTE and BtCatB. It is well established that the primary determinant of specificity for papain and cathepsins B and L is the S_2_ subsite [12,44-47]. Hydrophobic residues are preferred at the P_2_ position of substrates for papain and cathepsin L, but cathepsin B also accepts basic residues there. This difference is due to the presence of a glutamic acid at S_2_ of cathepsin B [12,46-48]. Covalent docking studies using the homology model of CPB2.8∆CTE reveal that the semicarbazone **CP229988** and the thiosemicarbazone **CP247129** have strict preference for the S_1_ to S_2_ subsites. A tyrosin (Y210) is located at the bottom of S_2_, which results in a much larger hydrophobic S_2_ subsite in the CPB2.8∆CTE enzyme compared with BtCatB. This is consistent with the finding that peptides containing hydrophobic amino acids at the P_1_ position, with hydrophobic and basic amino acids at P_2_ and P_3_, respectively, were resistant to hydrolysis by CPB2.8∆CTE but nevertheless had affinities in the nanomolar range [49]. Conversely, compounds CP229988 and CP247129 displayed only poor docking scores when covalently docked into the X-ray structure of BtCatB. These results suggest that specific inhibition of this cathepsin B relies on the primed subsite of the enzyme [50]. Two histidines located side by side in a large occluding loop form an area of strong positive charge in the S2’ subsite of BtCatB, which can be addressed by inhibitors with a negatively charged C-terminal group. In addition, the deep S1’ subsite of BtCatB prefers large hydrophobic residues of an inhibitor while cathepsin L has an opposite trend, favoring amino acids with small or long but non-branched site chains [51]. These observed differences in binding interactions and the corresponding difference in covalent docking ranks provide a cogent rationale for the observed lack in the BtCatB inhibitory activity of **CP247129**, **CP229988** and the triazine nitrile **CP241026**.

An encouraging result to emerge from this study was the discovery of three new non-peptide scaffolds of competitive inhibitors of *L. mexicana* CPB2.8∆CTE, with *K*_i_ values ranging from 5 nM to 570 nM. The covalent docking studies have provided an understanding of the importance of the determinants of inhibitory activity in CPB2.8∆CTE, and, as a result, we found that the docking ranks paralleled the activities on a qualitative level. Thus, this methodology could be employed as a guide in selecting new molecules.

Semicarbazones, thiosemicarbazones and triazine nitriles are warhead-types of compound groups already known to contain cysteine protease inhibitors. However, the four lead compounds identified by this study are novel inhibitors, with the semicarbazone **CP229988** being shown to have good activity and high specificity. Further research is needed to elucidate whether the potent inhibition in the biochemical assays is translated into efficacy also against the parasite itself in appropriate biological assays. The *K*_i_ values of lead compounds are, however, in the nanomolar range, which is a promising starting point for further lead optimization to generate compounds that could be candidate drugs.

## Experimental Section

### Reagents

All chemicals and the irreversible cysteine protease inhibitor E-64 (1-[*N*-[(L-3-trans-carboxyoxirane-2-carbonyl)-L-leucyl]amino]-4-guanidinobutane) were from Sigma-Aldrich Inc., St. Louis, MO, USA, unless stated otherwise. The Z-F-R-AMC substrate was from Bachem. 

### Homology protein modelling

The sequence of mature *L. mexicana* CPB2.8∆CTE was used to search the Brookhaven Protein Data Bank (PDB). To build the homology model, the crystal structure of cruzain in complex with the irreversible fluoromethyl ketone inhibitor Mor-Leu-Hpq was used as the template: PDB entry ID 1EWP [28]. Homology models were calculated using the program Modeller implemented in the Insight II software package (Accelrys Inc. San Diego, CA, USA) [52]. All calculations were carried out under default conditions. For the alignment of the CPB2.8∆CTE sequence to the template, the BLOSUM 62 matrix implemented in Modeller’s ALIGN123 module was taken. Four homology models were generated using the default conditions with the highest optimization level, and subsequently four additional structures were generated with a high loop refinement for each of the four initial homology models. In summary, 16 homology models were built to ensure the generation of the highest possible structure quality. The corresponding model with the lowest value of PDF violations in combination with the lowest energy value was selected for validation. The quality of the models was validated using the ProStat and Profiles3D method implemented in the Insight II software package. Disulfide bridges were checked and assigned manually using the Sybyl 6.8 software package (Tripos Inc., St. Louis, MO, USA).

### Enzyme source

A major cysteine protease (CPB) of *L. mexicana* that is predominantly expressed in the amastigote life cycle form that causes disease in mammals was overexpressed in *Escherichia coli*. The CPB enzyme was expressed as an inactive pro-form lacking the characteristic C-terminal extension, designated CPB2.8∆CTE. Purification from inclusion bodies to apparent homogeneity and activation of the recombinant enzyme was described previously [21]. The enzyme concentration determined by active site titration with E-64 was found to be 6 μM. The portion of active enzyme recovered was approximately 30% of the total recombinant enzyme [25]. Bovine cathepsin B (BtCatB, EC 3.4.22.1) was obtained from Sigma-Aldrich Inc., St. Louis, MO, USA.

### Screening library

The HTS library used against CPB2.8∆CTE and bovine cathepsin B (BtCatB) was a collection of 74,339 small molecules acquired from commercial vendors (Asinex, Ltd., Moscow, Russia; Akos Consulting & Solutions GmbH, Basel, Switzerland; ChemBridge Corporation, San Diego, CA; Chemical Diversity Labs, Inc., San Diego, CA; Enamine, Ltd., Kiev, Ukraine; InterBioScreen, Moscow, Russia; LifeChemicals, Inc., Burlington, ON, Canada; Maybridge, Cambridge, United Kingdom; Otava, Kiev, Ukraine; Specs, Delft, Netherlands; TimTec Corp., Newark, NJ; and Vitas-M Laboratory, Ltd., Moscow, Russia). The 74,339 compounds were selected from a 2 million vendor ISIS database (Symyx Technologies, Inc., Sunnyvale, CA) using the solutions module implemented within the software tool SYBYL, version 6.8 (Tripos, Inc., St. Louis, MO, USA). Molecules that contained atoms other than C, O, H, N, S, P, F Cl, Br, or I, which had a molecular mass of >450 Da or which possessed more than eight rotatable bonds were removed from the data set. Filtering tasks were done using the CACTVS software package [53]. The purity of all screening compounds used was >90%. 

### Chemicals

**CP247128** was ordered from InterBioScreen, Moscow, Russia; the IUPAC name is 2-phenyl-1H-indole-3-carbaldehyde thiosemicarbazone (purity of >95%); **CP247129** was also ordered from InterBioScreen; the IUPAC name is 5-(4-methoxy-phenyl)-2-furanyl-2-carbaldehyde thiosemicarbazone (purity of >95%); **CP241026** was ordered from Vitas-M Laboratory, Ltd., Moscow, Russia; the IUPAC name is 4-(cyclohexylamino)-6-[(2-methoxy-3-pyridinyl)amino]-1,3,5-triazine-2-carbonitrile (purity of >95%). **CP229988** was ordered from Chemical Diversity Labs, Inc., San Diego, CA; the IUPAC name is 3-[(1H-benzimidazol-2-yl)-2H-1-benzopyran-2-ylidene]-2-carbaldehyde semicarbazone (purity of >95%). 

### Determination of IC_50_ values

The activities of CPB2.8∆CTE and BtCatB were measured in a homogeneous fluorescence endpoint assay using Z-F-R-AMC as substrate, modified from published methods [17,54]. The fluorophore AMC (7-amino-4-methylcoumarin) is released by active cysteine protease, and can be detected at 360 nm excitation and 465 nm emission. The assay was optimized for automated high throughput screening on a Biomek FX workstation (Beckman Coulter). All assays were carried out in black 384 well plates at room temperature (22°C). Each plate contained 24 controls in the 23rd and 24th rows of the plate, including 8 positive controls and 8 blanks (without enzyme) as well as 8 inhibitor controls. E-64 was used as standard inhibitor. A final concentration of 10 µM of E-64 resulted in a complete inhibition of protease activity. Test compounds available as 10 mM stock solutions in DMSO were diluted further in DMSO, except for the last 1:10 step, which was done in H_2_O to lower the DMSO concentration in the assay. Final concentrations of the test compounds from 30 µM to 0.3 nM were used. For the *L. mexicana* CPB2.8∆CTE assay, 5 µl test compound or 5 µl DMSO in acetate buffer (10% DMSO) was added to each well of the microtiter plate. Subsequently, 25 µl of a premix containing sodium acetate buffer (final concentration [f.c.] 100 mM), titriplex II (f.c. 1 mM) (Merck, Darmstadt, Germany), DTT (f.c. 10 mM) (Carl Roth GmbH, Karlsruhe, Germany), triton (f.c. 0.01%) (Merck, Darmstadt, Germany), BSA (f.c. 0.1 mg/ml) (PAA Laboratories GmbH, Pasching, Austria) and enzyme was added to each well, except the wells for the blanks, which include the premix without enzyme. After an incubation time of 10 min, the reaction was started by addition of substrate (20 µl Z-F-R-AMC; f.c. 20 µM). The reaction was stopped after 35 min incubation at room temperature by addition of 10 µl inhibitor E64 (f.c. 16.7 µM) and enzyme activity was measured with a SpectraFluorPlus (Tecan Inc., Durham, NC) plate reader using excitation at 360 nm with emission at 465 nm. The assay procedure for BtCatB was the same as for the CPB2.8∆CTE assay with the exceptions of the final concentration of the substrate Z-F-R-AMC (100 µM) and the stop of the assay using a stop buffer containing sodium acetate and sodium chlorine acetate (f.c. 41.7 mM each). 

The percentage enzyme activity was calculated using the formula (V-B)/(C-B) x 100%, with V being the absorbance of the assay containing the test compound, B being the absorbance of the negative control (‘blank’) and C being the absorbance of the positive control. In primary screening, all compounds of the screening library were tested at 10 µM in double point measurements. Z´ factors, which describe the quality of an assay, were in the range of 0.85-0.99. The Z´ factor is defined in terms of four parameters: the means and standard deviations of both the positive (p) and negative (n) controls: Z´= 1-((3 x (STDEVp + STDEVn))/(MEANp - MEANn)) [55].

The theoretical hit limit for a HTS primary screen can be calculated as MEAN + 3 x STDEV of inhibitions from diverse compounds. If a normal distribution for inhibition is expected, 99.73% of inactive compounds are within this limit. In our case, based on a preliminary screen of >3000 diverse compounds, the hit limit was determined between 12.8% and 22.9% inhibition. For practical reasons, screening hit limits are normally set above this limit, aiming at approximately 0.2-0.4% hits from a diverse library. Therefore, 50% inhibition at 10 µM compound concentration compared to the positive controls was considered a hit compound and were subjected to hit verification (IC_50_ determination from liquid compound stock) using CPB2.8∆CTE and BtCatB to get the activity profile of the compounds. Hit confirmation (IC_50_ determination from freshly dissolved solid compound stock) was performed for all compounds with an IC_50_ ≤ 30 µM on CPB2.8∆CTE. Compound concentrations used for IC_50_ determinations were as follows: 30 µM, 10 µM, 3 µM, 1 µM, 0.3 µM, 0.1 µM, 0.03 µM, 0.01 µM, 0.003 µM, 0.001 µM, and 0.0003 µM. Enzyme activities were expressed as percentages of residual activity compared with an uninhibited control and were plotted versus increasing inhibitor concentrations. IC_50_ values were calculated using the four-parameter equation model 205 and the option “unlock” from the XLfit add-in (IDBS, Guildford, United Kingdom) in Excel (Microsoft Corporation, Redmond, WA). All values are mean values from at least three independent assays to ensure statistically significant results. 

### Calculation of *K*_i_ values

*K*_i_ values of the proposed lead compounds were calculated using the Cheng-Prusoff equation (*K*_i_ = IC_50_/(1 + [S]/K_m_) with [S] = 20 μM and K_m_ = 21.3 μM for CPB2.8∆CTE[32]. It is assumed that the leads are reversible competitive inhibitors.

### Compound solubility measurements

A 2-fold serial dilution of the compounds was performed (0.5 to 500 µM) in DMSO (Acros Organics, Fischer Scientific, Morris Plains, NJ) and added in duplicate to 96-well microtiter plates. Phosphate-buffered saline (PBS; pH 7.4) was added to give a total volume of 200 µl and a final DMSO concentration of 5% (vol/vol) in all wells. The plates were incubated at room temperature for 22 min, and the relative solubilities of the compounds were determined by measuring forward-scattered light using a NEPHELOstar laser-based microplate nephelometer (BMG Labtech GmbH, Offenburg, Germany). Wells containing only buffer and 5% (vol/vol) DMSO were used as controls. Data analysis was carried out using Excel (Microsoft Corporation, Redmond, WA). 

### NMR and LC-MS analyses

Compound purity and molecular mass were confirmed by liquid chromatography-mass spectrometry (LCMS) experiments, which were performed on an Agilent LC7 MSD (Mass Selective Detector) 1100 LC-MS (Agilent Technologies, Santa Clara, CA). The liquid chromatography conditions were as follows: a Zorbax SB (stable bound) C18 column, 1.8-µm particle size, column dimensions of 4.6 by 30 mm, 0.1 µl/min flow rate, gradient of 10 to 100% eluent B over 3 min (eluent A was 95:5 H_2_O to CH_3_CN supplemented with 0.1% formic acid, and eluent B was CH_3_CN), and a column temperature of 313 K. MS detection was performed using an MSD 1100 electrospray ionization (ESI) and atmospheric pressure ionization (API) (Agilent Technologies, Santa Clara, CA) single quadrupole mass spectrometer. Nuclear magnetic resonance (NMR) spectroscopy was carried out on a Bruker Avance DRX 400 MHz NMR spectrometer (Bruker AXS, Madison, WI). 

### Covalent docking

Covalent docking was performed as previously described [24]. Version 5.0.1 of the GOLD docking suite (The Cambridge Crystallographic Data Centre) was used for covalent docking studies [56,57]. The resulting docking solutions were ranked based on the selected scoring function. In covalent mode, the program assumed that there is just one atom linking the ligand to the protein. Both protein and ligand files were set up with the link atom included. During docking runs, the link atom in the ligand is forced to fit onto the link atom in the protein. In order to ensure that the geometry of the bound ligand was correct, an angle-bending energy term for the link atom was included in the calculation of the fitness score [56]. The above-described covalent docking mode implemented in GOLD was applied for all docking runs using standard default settings. The scoring function GoldScore was used in its modified version for covalent docking. Protein structures were prepared according to the GOLD user manual and ligand structures by application of a CACTVS-based script [58]. If the electrophilic carbon atom of a warhead represented a prochiral center, both possible stereoisomers for the resulting compound were generated and treated as distinct ligands for the further processing. The sulfur atom of the C26 residue in the *L. mexicana* CPB2.8∆CTE homology model and the sulfur atom of the C29 residue in the BtCatB X-ray structure (PDB ID 1QDQ) were defined as the link atoms for the covalent bond. The three-dimensional (3D) illustrations of the covalent docking results depicted in Figure 4 were generated using MOLCAD [34]. 
